# Salient Distractors Can Induce Saccade Adaptation

**DOI:** 10.1155/2014/585792

**Published:** 2014-04-30

**Authors:** Afsheen Khan, Sally A. McFadden, Mark Harwood, Josh Wallman

**Affiliations:** ^1^Department of Biology, City College of New York, Marshak Science Building, Room 526, New York, NY 10031, USA; ^2^School of Psychology, The University of Newcastle, Callaghan, NSW 2308, Australia

## Abstract

When saccadic eye movements consistently fail to land on their intended target, saccade accuracy is maintained by gradually adapting the movement size of successive saccades. The proposed error signal for saccade adaptation has been based on the distance between where the eye lands and the visual target (retinal error). We studied whether the error signal could alternatively be based on the distance between the predicted and actual locus of attention after the saccade. Unlike conventional adaptation experiments that surreptitiously displace the target once a saccade is initiated towards it, we instead attempted to draw attention away from the target by briefly presenting salient distractor images on one side of the target after the saccade. To test whether less salient, more predictable distractors would induce less adaptation, we separately used fixed random noise distractors. We found that both visual attention distractors were able to induce a small degree of downward saccade adaptation but significantly more to the more salient distractors. As in conventional adaptation experiments, upward adaptation was less effective and salient distractors did not significantly increase amplitudes. We conclude that the locus of attention after the saccade can act as an error signal for saccade adaptation.

## 1. Introduction

Saccades are the rapid eye movements that we use to explore the visual world. They typically last a few tens of milliseconds, so the ongoing movement cannot use visual feedback for guidance [1]. This means that the size and direction of the saccade are planned before the eyes move and accuracy is maintained based on error signal(s) related to the consequence(s) of their movement.

An adaptive control mechanism ensures saccade accuracy in the face of changes in motor dynamics due to daily fatigue, aging, or pathology. When extraocular muscle impairment or weakness occurs, the initial large targeting errors diminish over time, meaning that the motor planning is able to adapt and restore saccade accuracy [2–5]. On a much more rapid time scale in the laboratory, surreptitiously displacing a target while the eye is in mid-flight with an intrasaccadic step (when vision is impaired) tricks the oculomotor system into thinking that the saccade had been inaccurate because, when the saccade lands, the target is no longer on the fovea. If this occurs consistently, the oculomotor system gradually adjusts its saccade amplitude to partially compensate for the imposed error [6]. Despite the observer being unaware of the displacement, their saccades land progressively closer to the displaced position rather than the initial position of the target.

There are several possibilities as to what constitutes the error signal driving such saccade adaptation. Although a simple proposal would be for the oculomotor system to track the relative direction of the primary and the subsequent small corrective saccades needed to foveate the target [7], it appears that adaptation can still take place when there are very few corrective saccades made [8], and saccades can be adapted in the opposite direction to the correctives [9]. Retinal error, which is the distance of the target from the fovea after each saccade, has also been proposed as an alternative error signal. Since saccade amplitude can be induced to decrease when the target is displaced backwards and increase when the intrasaccadic step is forward, the direction and distance of the target from the fovea when the saccade lands could provide a simple possible error signal to guide saccade adaptation ([8, 10, 11], reviewed in [12]). However, a direct test of the potency of retinal error alone in driving saccade adaptation in the absence of actual intrasaccadic steps showed that it is much weaker than that provided when the predicted target position was changed [13]. Earlier evidence that adaptation is not driven by the size of the retinal error with respect to the target but by the retinal error with respect to the predicted location was provided by Bahcall and Kowler [9]. They demonstrated that there was no saccade adaptation when subjects were instructed to make saccades not to the target but to a location that was 75% of the distance to the target, showing that the retinal error at the end of the saccade to the target alone was not sufficient to cause saccade adaptation. However, with the same saccade goal of 75% of target distance, adaptation was seen when the target did make intrasaccadic steps (saccade amplitudes reduced despite the stepped target remaining beyond the saccade landing position), suggesting that it is the retinal error with respect to the predicted location that is the key. Mathematical models of such “sensory prediction errors” are standard in arm movement adaptation and have also been formulated for saccades ([14]; for reviews see [15, 16]).

We propose that the locus of attention might offer an alternative, or complementary, framework to account for saccade adaptation. At one level, Posner [17] operationally defined attention with respect to expectation (prediction) of cue location being valid. More pertinently, recent evidence shows that attention is predictively allocated before the saccade, to the retinal location in which a secondary target will appear after the saccade is complete [18]. Rolfs and colleagues argued that this predictive remapping of attention facilitates the secondary saccade planning. Attention leaves a “retinotopic trace”: the retinal position of attention before a saccade can be seen in the same retinal location after a saccade [19]. Hence, if Rolfs's predictively remapped attention coincides with the retinal location of target after saccade, this might also help explain the perceived stability of the world across saccades to stationary targets [20, 21].

Other work in our lab [22, 23] led to a very similar “placeholder hypothesis” of how attention might keep track of target positions across saccades, in particular, by providing a potential error signal for saccade adaptation. It was partly motivated by another useful feature of attention: selection. How in the real, visually cluttered world might saccade adaptation keep track of “the” error signal? Since attention helps to select the target before the saccade, with presaccadic shifts of attention to the target location [24–27], we hypothesized that attention could act as a placeholder for the target and that mismatches when comparing pre- and postsaccade loci of attention could act as an error signal for adaptation. To account for the Bahcall and Kowler partway paradigm above, our hypothetical comparison has to be between the predicted and actual postsaccadic locus of attention. That is, the predicted postsaccadic attention locus would be dissociated from the fovea when a saccade lands partway and instead be found at the presaccadic locus of attention (the original target location) towards which a secondary saccade is being planned. When the target is not stepped intrasaccadically there is no mismatch between predicted and actual postsaccadic attention locus, and no adaptation occurs. When the target is stepped backwards, saccades adapt downwards due to the mismatch, despite the target still being more eccentric to the fovea. Although this prediction necessitates some kind of corollary discharge signal and attention can be spatiotopically encoded [28] given the recent evidence [29] and preceding arguments [18, 19], we believe that an attentional comparison in retinotopic coordinates is more tenable.

How is this different from sensory prediction errors? Have we not just substituted predicted and actual “attention locus” for predicted and actual “target location”? Dissociating these was the prime motivation behind the current set of experiments. Abrupt onset distractors can attract attention [30] away from saccade targets [31]. We proposed to use sudden onset salient distractors immediately after saccades to attempt to automatically draw the attention locus away from the extant, unmoved target during the critical window for saccade adaptation (<250 ms after saccade, [32–34]). If predicted and actual target location were critical, no adaptation should occur. If attention locus has a potential role then we conjecture that these distractors would create a mismatch between the pre- and postsaccadic locus of attention, even if the saccade had been accurate, and induce saccade adaptation in the direction of the attentional shift.

A secondary motivation was to dissociate between attention affecting saccade adaptation purely at a selection level and our hypothesis that the locus of attention may be directly involved in the computation of the error signal itself. Ditterich et al. [35] also argued for a comparator model based on attention focus, but attention was primarily invoked to select a snapshot of visual information before the movement (at the locus of the presaccadic attention shift) and then to compare that directly to the reafferent visual information on saccade landing. Briefly, they had two target conditions: a 0.3 deg fixation cross and a 4.8 deg ring, both on backgrounds consisting of random arrays of ellipses. Shifting the background during the saccade induced adaptation when subjects attended to the ring but not when attending to the cross. They argued that the attention focus selected the background in the ring condition, leading to adaptation, but only the unmoved small target in the cross condition, leading to no adaptation.

We argued that, according to the Ditterich scheme, if the visual information after the saccade was not shifted, but simply extra elements were added, there would be no error signal as there would be no mismatch to the information selected prior to the saccade. Consistent with this, we have previously shown that a distractor appearing after a saccade does not invoke saccade adaptation in the direction of the distractor [36]; instead, the adaptation mechanism is able to select only the relevant target information for computation of its error signal. The difference between our current hypothesis and that of Ditterich et al. [35] is that theirs relied on a visual comparison and ours relies on a position of attention comparison. Our previous work [36] does not differentiate between these two. In that study, the small distractor (0.5 deg) had the same size and salience as the target, always appeared at the same location after saccade, and stayed on for the rest of the trial. We suspect that this repetitive stimulus had a very weak attention-attracting effect [37], and therefore the locus of attention was unmoved by the appearance of the distractor after saccade. In the current set of experiments, we used many different distractors, which changed regularly and were much larger and more visually salient than the target itself. We show here that despite subjects dutifully making saccades to the targets, not to the distractors, saccade gain was influenced by the location and salience of the distractor.

## 2. Methods

### 2.1. Subjects

Two experienced and twelve naïve subjects participated in Experiment 1 (7 female, 7 male) including two of the authors. The same two experienced and eight (new) naïve subjects participated in Experiment 2 (4 female, 6 male) including two of the authors. Naïve subjects were recruited from the City College Psychology Department and received course credit for their participation. All subjects had either normal or corrected vision. The Institutional Review Board of the City College of NY approved the experimental protocol, and all subjects signed consent forms before participation.

### 2.2. Equipment

The stimuli were generated and under the control of an application written in LabView (National Instruments). Stimuli were displayed on a 22 inch Compaq color, CRT monitor with a vertical refresh rate of 160 Hz. Subjects observed the stimuli while seated 57 cm away from the monitor, in an otherwise darkened room.

### 2.3. Eye Movement Recording and Analysis

Eye movements were recorded using an infrared video eye-tracking system (EyeLink, SR Research Ltd, Mississauga, Ontario, Canada), which sampled pupil position at 1000 Hz. The pixel-to-degrees calibration of the eye tracker was based on having each subject fixate a 0.1 deg target at locations taken from a 9-point rectangular grid. During the experiments, the distractor onset was triggered when a saccade was detected based on a velocity threshold criterion of 30°/sec. During analysis, saccade start- and endpoints were defined by a 10°/sec velocity threshold. Subjects' head position was held steady during the experiment by use of a chin and forehead rest.

### 2.4. Procedure

Each subject's task was to follow a target spot (0.1° in diameter), while they were tested consecutively in three phases. The first phase (preadaptation) and the last phase (postadaptation) were identical and each consisted of 100 trials to assess normal saccade gain to a target step under open-loop conditions. The intervening 2nd phase (adaptation) consisted of 250 trials in which attentional distractors were present.

In each trial during the preadaptation and postadaptation phases, the target spot randomly stepped left or right, initially from a central fixation position and subsequently from its previous target location on successive trials. During every experiment, the target moved only along the horizontal meridian and never moved further than 13° from the center of the screen. The target step size randomly varied between 7° to 9° and occurred 900–1400 ms after the end of the previous trial. As soon as a saccade was detected based on the velocity threshold, the target spot disappeared for 500 ms and then reappeared in the same location until the end of the trial. The complete trial length was 1800 ms. In theory, this brief removal of the target once the saccade was underway should prevent any immediate postsaccadic visual error from mitigating any adaptation that had been previously established in the postadaptation phase. The change in saccade gain between the preadaptation and the postadaptation phases was the primary measure used to determine the effect of the adaptation trials on saccade gain.

The adaptation phase consisted of 250 distractor trials in which the target spot stepped the same as in the other two phases, but when the saccade occurred there were two differences: first, the target remained continuously on until the end of the trial (again trials were 1800 ms long); second, additional salient distractors were presented either on the near side of the target (referred to as adapt-down condition) or the far side of the target (referred to as adapt-up condition) during and after the saccade in Experiment 1. In Experiment 2, the distractors were always presented on the near side of the target. The details of distractor type and location varied in both experiments as described below. In addition, there were 25 interleaved trials in which the target disappeared upon saccade detection identical to the pre- and postadaptation trials, and hence, there were no distractors presented in these trials. In both experiments, the different adaptation conditions (distractor location or type) were performed in separate sessions at least one day apart, the order of which was randomly selected.

### 2.5. Experiment  1: The Effect of Salient Visual Distractors in the Adapt-Down and Adapt-Up Condition

**Figure 1:**
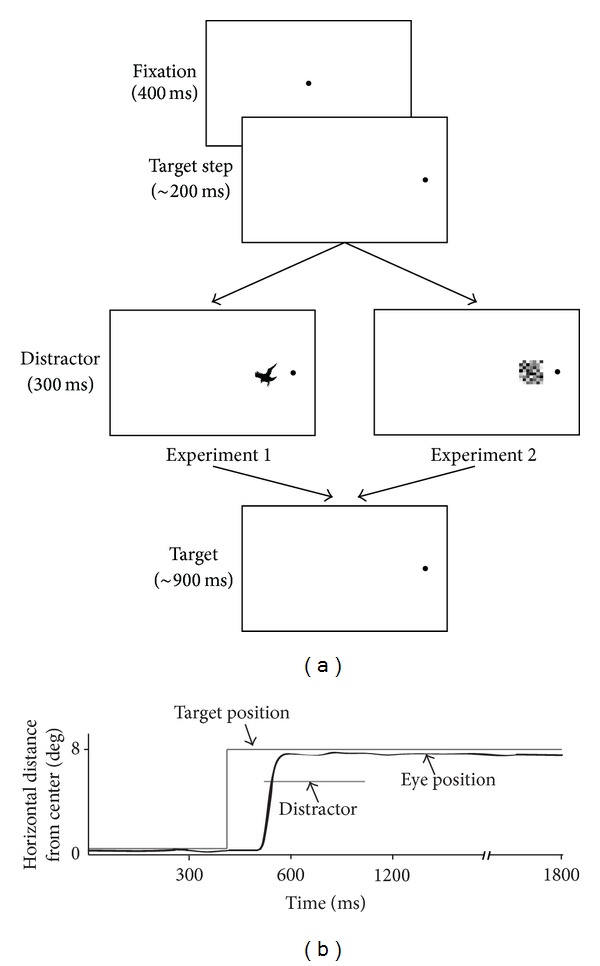
(a) Diagram of screen appearance and timing during the downward adaptation trials for the two experiments. Trials in both experiments consisted of a fixation, which stepped to the left or right after a brief variable delay (900–1400 ms, of which only 400 ms was recorded). Diagram shows a rightward step example only. Upon saccade detection, a visual distractor was presented for 300 ms, after which it disappeared leaving only the target on the screen in the stepped location. An example of both types of distractors used in both experiments is shown (drawn approximately to scale). See text for a description of the full set of distractors used in each experiment. (b) A schematic of target, distractor, and eye position during an adaptation trial. After the target step, the eye follows with a saccade, upon which time a distractor is presented a short distance from the target, which remains at the stepped location for the remainder of the trial.

The aim in this experiment was to use salient visual stimuli to attract attention away from the target position during the postsaccadic window so that an attentional error signal might be generated. To do this, during the adaptation phase, when the subject made a saccade to the target (0.1° red spot on a white background which stepped 7–9° randomly to the left or the right), one of 35 different distractor images was randomly selected to appear consistently centered at 3° on the inner side of the target (i.e., at 62.5% of the initial target step, on average) for 300 ms. This timing was selected to allow sufficient time for attentional disengagement with the target and brief reallocation to the visual distractor. It should be noted that, since the spot was left on and the trial lasted for 1.8 sec, there were plenty of attentional resources allocated to the target location with which our early attentional disruption had to compete. The large number of possible distractors was in order for the distractors to remain salient and to prevent habituation to the distractors so that they might continue to draw attention over the 250 adaptation trials. Each image was presented no more than 8 times. Distractors consisted of birds, other animals, anime characters, recognizable objects, and popular cartoon characters. Each image was 2.1° in horizontal width and varied in height between 1.5° and 3.5° (an example is shown in Figure 1). In the adapt-down condition of Experiment 1, we asked whether a strong visual attentional signal located on the near side of the target might also be able to compete with the target to bias saccades to decrease their amplitudes. The adapt-up condition of this experiment was identical to the adapt-down condition, except that the visual distractors were centered 3° on the far side of the target spot. The same subjects were tested in both the adapt-down and adapt-up conditions, to compare the effectiveness of attentional location to cause amplitude decreases versus increases, respectively.

### 2.6. Experiment  2: The Effect of Nonsalient Distractors on the Near Side of the Target

In Experiment 2, we asked whether a distractor that was less attention grabbing than those used in Experiment 1 would cause an equal or diminished magnitude of adaptation. This experiment was identical to the adapt-down condition of Experiment 1, except that the visual distractor used was a random noise pattern that was similarly 2.1° in horizontal width and also varied in height between 1.5° and 3.5°. This will be referred to as the “nonsalient condition.” The same subjects also performed a task that was identical to the adapt-down condition of Experiment 1 (to be referred to as the “salient condition”) in order to compare the magnitude of adaptation to the salient distractor images with that of the nonsalient, repetitive noise pattern.

### 2.7. Data Analysis

The data presented here are the changes in the gain of the primary saccade during the pre- and postadaptation phases, which were the open-loop saccade periods before and after the attention distractor trials. All trials were previewed in a custom graphical interface (Matlab, The Mathworks, Natick, MA). Statistical tests for individual experiments were based on paired *t*-tests. The raw data are shown together with a 20 point moving average calculated separately for each phase of the experiment. Saccade amplitudes elicited when the distractor was on the near or the far side of the target were compared using repeated measures ANOVA within individual subjects.

## 3. Results

In brief, we found that a salient visual stimulus displayed on the near side of the target after the saccade was underway caused a decrease in saccade gain. The magnitude of adaptation was reduced when a nonsalient, neutral distractor stimulus was used in all of the adapt trials instead of the salient visual images. The salient images in Experiment 1 were used to increase the attraction of attention away from the continuously visible, unchanging target and towards the locus of the distractor. These findings suggest that a discrepancy between the locus of attention and the fovea can provide an error signal to drive saccade adaptation. Moreover, when the distractor is not salient, the magnitude of adaptation that the distractor is able to induce is diminished.

### 3.1. Experiment  1: Salient Images Can Change the Saccade Gain

**Figure 2:**
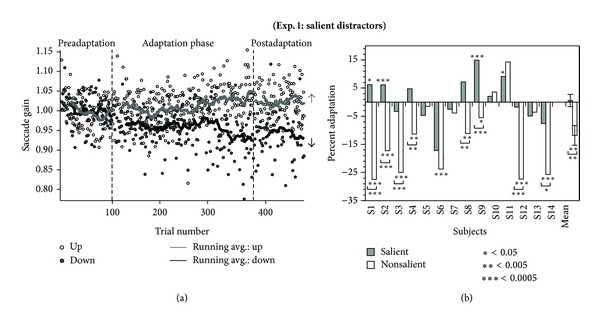
(a) Saccade gain data of subject S1 during Experiment 1 in which distractors consisted of salient images. Gain decreased gradually in the adapt-down condition (Experiment 1(a), closed circles, black curves) and displayed a significant decrease in gain of the postadaptation phase from the preadaptation phase. The adapt-up condition did not demonstrate any significant change (Experiment 1(b), open circles, gray curves). (b) Percentage adaptation (calculated as the difference between the pre- and postadaptation phases relative to the percent of distance of the distractor from the target) for each subject. Mean and standard error across all subjects are shown on the right. The asterisks below or above the bars represent significance between the pre- and postadaption phase in an individual experiment, the asterisks below a square bracket represent the significance between the adaptation in the adapt-down compared to adapt-up condition.

An example of the raw data from one subject in both the adapt-down and adapt-up conditions of Experiment 1 is shown in Figure 2(a). It is evident that in the adapt-down condition the saccade gain declined throughout the adaptation portion of the experiment. We found that, when the visual distractor was on the near side of the target, the postsaccadic gain decreased significantly in 9 out of 14 subjects (*P* < 0.05 in 2 cases and *P* < 0.01 in 7 cases). We calculated the percentage adaptation, which was the difference in saccade gains between the pre- and postadaptation phases divided by the maximum gain change possible (calculated as the difference between the pre- and postadaptation phases relative to the percent distance of the distractor from the target). The average decrease for all subjects was 11.8 ± 3.5% after 250 adaptation trials (one-way repeated measures ANOVA, *F* = 11.4, *P* = 0.005, Figure 2(b)).

In the adapt-up condition when the distractor was presented on the far side of the target, the distractors were less effective in increasing the gain than decreasing it (Figure 2). Gain increased significantly in only four subjects (one-tailed *t*-tests). Across subjects, the average increase in gain between the pre- and postadaptation phases was 0.6 ± 2.2% which was not significant (one-way repeated measures ANOVA, *F* = 0.0876, *P* = 0.77; Figure 2(b)). However, the interaction between phase (before versus after) and adaptation direction (down versus up) across all subjects was significantly different (repeated measures ANOVA, *F* = 13.6, *P* = 0.003).

### 3.2. Experiment  2: Nonsalient Random Noise on the Near Side of the Target Caused Less of a Decrease in Saccade Gain

**Figure 3:**
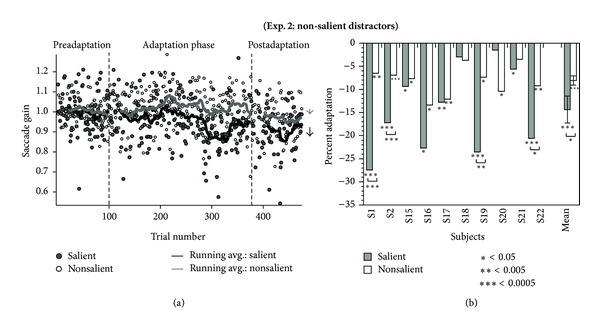
(a) Saccade gain data of subject S2 during Experiment 2 in which distractors consisted of a random noise patch (nonsalient) in Experiment 2(a) and salient images in Experiment 2(b). Gain decreased gradually in the presence of the random noise distractor (Experiment 2(a), open circles, grey curves) and displayed a significant decrease in gain of the postadaptation phase from the preadaptation phase. The salient distractors decreased gain even more rapidly and demonstrated a significant change (Experiment 2(b), closed circles, black curves). (b) Percentage adaptation (calculated as the difference between the pre- and postadaptation phases relative to the percent distance of the distractor from the target) for each subject. Mean and standard error across all subjects are shown on the right. The asterisks below each bar represent significance between the pre- and postadaptation phase in an individual experiment. The asterisks below a bracket represent the significance between the adaptation in the salient distractor and nonsalient distractor condition.

A nonsalient, random noise distractor alone was able to decrease saccade gain when presented on the near side of the target, as can be seen by the raw data from a sample subject in Figure 3(a). Comparing the saccade gain during the preadaptation and postadaptation phases, we found a significant decrease in eight out of the ten subjects in the nonsalient condition. The average decrease was 8.1 ± 1.0% (one-way repeated measures ANOVA, *F* = 60.9, *P* = 0.00003, significant). The overall decrease in gain between the pre- and postadaptation phases for the salient condition was 14.4 ± 2.9% (one-way repeated measures ANOVA, *F* = 24.2, *P* = 0.0008). Only two subjects (S1 and S2) were common to both experiments, and hence there were small differences in the average gain decrease in the salient condition of Experiment 1 (11.8%). Both the random noise and the salient distractors caused a gain decrease. The magnitude of gain decrease between the pre- and postadaptation conditions was significantly different between the two distractor types for 4 of the 10 subjects (Figure 3(b)). The overall effect of distractor type was significant across all subjects as demonstrated by the interaction between the phase (preadaptation and postadaptation) and distractor type (salient and nonsalient) (repeated measures two-way ANOVA, *F* = 4.99, *P* = 0.05).

### 3.3. Corrective Saccades

Corrective saccades to the distractors cannot explain our observed gain changes. Because we are testing the hypothesis that the presumed change in the postsaccadic locus of attention is responsible for the gain changes, we needed to assess whether we had inadvertently provided other cues, in particular, whether subjects made secondary saccades to the attentional distractor and then made a third saccade back to the target when the distractor disappeared. If this occurred during the adaptation phase, the direction of the secondary saccade might function as an error signal, as corrective saccades have been hypothesized to do during normal saccade adaptation. In our experiment, although hypothetical saccades to the distractors would be followed by ones in the opposite direction, this could still function as a motoric error signal, because the saccade toward the distractors would occur earlier within the postsaccadic window that is most responsive to error signals. To evaluate this possibility, we assessed the number of secondary saccades to the distractors made during the adaptation phase of the experiment. In the adapt-down conditions of both experiments, corrective saccades made were considered to have been made to the distractor if they were downwards and resulted in eye gaze landing closer to the distractor than the target. In the adapt-up condition of Experiment 1, corrective saccades were considered to the distractor if they were forward and resulted in gaze landing closer to the distractor than the target. During the adaptation phase of Experiment 1, when the distractors were presented on the near side of the target (adapt-down condition), saccades were made to the distractor on average in 1.6% of the total number of valid trials (Table 1). Similarly, on average, during the adaptation phase of the adapt-up condition subjects made corrective saccades to the distractor in 7.3% of the total number of valid trials. The correlation between the number of corrective saccades to the distractor and the change in saccade gain for the adapt-down condition was −0.09 (*P* = 0.76) and was 0.24 (*P* = 0.40) for the adapt-up condition. Hence, we found that the number of secondary saccades to the distractor was not correlated with the change in saccade gain, suggesting that the error signal is more likely to be related to the shift of attention than to incidence of corrective saccades. Additionally, no correlation between the change in saccade gain and the number of corrective saccades to the distractor was also found in Experiment 2. The correlation between the adaptation and the number of corrective saccades to the distractor for the adapt-down salient distractor condition was 0.12 (*P* = 0.73), and for the nonsalient distractor condition the correlation was 0.3 (*P* = 0.40) (in which on average 0.9% and 0.8% of the correctives were to the distractor in the adaptation phase, respectively) (Table 2).

## 4. Discussion

We find that salient, unpredictable image distractors appearing after a saccade can lead to saccade adaptation despite the target remaining visible and stationary. In both experiments, when distractors were consistently presented on the near side of the target during the adaptation phase, we found that saccade gain gradually decreased. As we decreased the salience and image unpredictability of the distractor, the magnitude of adaptation decreased (Figure 3). Even though the distance between the target and visual distractor was within the range of intrasaccadic step sizes shown to be most effective for saccade adaptation [38], the adaptation that we observed was smaller than that typical in conventional saccade adaptation experiments ([6], reviewed in [12], reviewed in [39]). The smaller magnitude of adaptation in our experiment however is not surprising considering that the target spot did not make any intrasaccadic movements and simply remained in its stepped location even after the saccade was made. Since a postsaccadic error must persist for >32 ms to affect adaptation [32, 34], although we may have attracted attention away from the fovea, it was in competition with multiple veridical cues associated with the target being located on the fovea, and the putative attentional shift was presumably transient, as it had to be shared between the distractor location and the target location at the fovea. The adaptation we found was similar to conventional saccade adaptation, in terms of the asymmetry of larger gain changes for downward adaptation than for upward adaptation.

### 4.1. Possible Error Signals for Saccade Adaptation

Possible error signals for saccade adaptation must incorporate prediction and selection mechanisms. Previously, either the motor error (i.e., corrective saccades) or the visual error (difference between the target position and the eyes landing position) was thought to drive adaptation [7]. We know now that corrective saccades are not necessary for saccade adaptation, suggesting that the error signal is visual [8]. However, it appears that it is not purely a sensory error, but rather is the difference between the actual retinal image of the target at the end of a saccade and the predicted retinal image after the saccade that primarily drives adaptation [9, 13, 35, 40, 41], and yet visual feedback of the target is not even required for saccade adaptation to occur [42]. In addition to suggesting that prediction was required in saccade adaptation, Ditterich et al. [35] invoked selective attention to explain some of their data. They argued that a large scale of attention coded their background stimulus along with the saccade target (see Section 1), such that the background influenced adaptation, and a small scale did not code the background, which therefore had no influence on adaptation. Similarly, we and others have found that the adaptive mechanism can selectively ignore shifts of the background when a target is present [43, 44] or that static backgrounds have no effect on adaptation [45].

Attention is intimately linked to prediction, selection, and saccades [17, 24–28, 35]. Here, we tested the novel hypothesis that the locus of attention can act as the error signal for adaptation, by attempting to consistently direct attention to an attention grabbing, postsaccadic distractor located a few degrees from the target. As detailed in Section 1, if we assume that the sudden onset distractor draws attention away from the target, it would lead to a mismatch between the predictively remapped locus of postsaccadic attention and the actual locus of distractor captured attention. We conjectured that this would lead to adaptation towards the distractor to reduce the mismatch. In our experiment, the target was still present postsaccadically and in the predicted retinal location. The fact that neither retinal error nor predictive error can explain our findings presents a challenge to current ideas for the error signal guiding saccade adaptation. We must emphasize, of course, that we are conjecturing as to the attention grabbing properties of our sudden onset, salient distractors. In future work we would like to include specific attention tasks to confirm these findings. However, as argued in Section 1, the addition of salient, unpredictable distractors after the saccade is a feasible means to distinguish between the existing evidence for attention acting on adaptation shown by Ditterich et al. [35] and our locus of attention hypothesis.

### 4.2. Ethology of Saccade Adaptation

Attention is likely to be used to maintain the accuracy of saccades since in the visually complex natural world overt attention generally follows covert attention to objects that attract our attention. Most saccade adaptation experiments however rely on very sparse visual stimuli that rarely require any target selection. This fails to reflect the continuous competition in the natural world for our limited resources. In the natural world we utilize covert attention to select our next focus of gaze [46]; therefore it is likely also involved in maintaining saccade accuracy as well. Although it has been shown that attention shifts and saccades can be dissociated [47, 48], it is unlikely that a saccade would be judged inaccurate if the saccade were made to what was being covertly attended in a crowded visual scene. It has been previously shown that only the attended visual information after a saccade is utilized in saccade adaptation [35]. Therefore, we propose that the locus of attention after a saccade is also integrated to deem whether a saccade is accurate or not.

For the purposes of saccade adaptation the oculomotor system has demonstrated the ability to select the target over other distractors presented on the screen [36]; however we demonstrated here that salient, unpredictable distractors are able to interfere with this target selection. The Madelain et al. [36] experiment was similar to the current study in that a distractor was presented after saccade initiation a short distance from the target (2° or 2.4°) but differed in that it was present for the remainder of the trial. More importantly, unlike the array of possible distractors used in the present experiment, the distractor in the Madelain et al. [36] experiment was not more salient than the target; indeed, the target and distractor were interchangeable between trials (the target was simply the first of the two colored spots to appear). Although explicit attentional tasks were not included in either study, it is likely that the distractors in the Madelain et al. [36] experiment had a weaker attention attracting effect than the visual distractors in Experiment 1 in the current study. Due to the predictable color and location of the distractor in Madelain et al. [36], they may even have prevented capturing attention (e.g., see [49, 50]). Therefore, the mere presence of a visual stimulus on the screen along with the target by itself will not elicit saccade adaptation [36] unless, as implied in our experiments, the distractor is salient and unpredictable enough to consistently attract attention.

To further test this relative salience and unpredictability explanation for the differences between our data and that of Madelain et al. [36], we compared varied and salient natural images to fixed, static noise patch distractors in Experiment 2 and found that the predictable noise patch distractors (but ones larger and more salient than the target) gave some, but weaker, adaptation than the set of salient distractors. Thus, between our two experiments and those of Madelain and colleagues we observe a spectrum of increasing predictability and decreasing salience of distractors leading to decreased levels of adaptation, with predictable distractors of equal salience to the target being effectively ignored by the adaptation machinery [36]. In the absence of an explicit attentional task, we conjecture that the locus of attention after the saccade is more strongly drawn away from the saccade target by larger or more unpredictable distractors and that this directly caused the differences in adaptation that we have observed.

### 4.3. Alternative Explanations

One might argue that the gain change occurs because the oculomotor system does not distinguish between the saccade target and the attentional distractors, despite their diversity and dissimilarity to the target. That is, the center of gravity of all the stimuli on the screen might be used for computing an error signal or adaptation occurs due to some averaging of the target and distractor locations such as in the global effect [51], albeit postsaccadically for our situation since the distractor was not present at the time of saccade onset. However, no global averaging of the target and distractor locations was found in a similar experiment where the distractor was similar in appearance to the target [36]. Under these conditions, despite a global stimulus configuration similar to ours, the oculomotor system effectively distinguished between the target and the distractor. Furthermore, if the purely visual signal of having more stimuli on one side of the fovea than on the other might act as an error signal to adjust the saccade gain in the directions shown here, it is surprising that such a small percentage of the secondary saccades (average of 1.6% for adapt-down condition in Experiment 1 and 7.3% for the adapt up condition) were made to the distractors, making it unlikely that they were regarded as the saccade target. Additionally, if the global effect were responsible for the saccade adaptation that we observe, it would be expected that the magnitude of the adaptation might be similar between upward and downward adaptation, and between the unpredictable/salient and the predictable/reduced salient conditions of Experiments 2. Instead it was found that the predictable distractors used in Experiment 2 produced a gain decrease that was about half of that produced in the more salient/less predictable condition.

### 4.4. Similar Characteristics to Conventional Saccade Adaptation

The gradual adaptation in our distractor paradigm was similar to adaptation elicited in conventional intrasaccadic paradigms. In Experiment 1 there was the same asymmetry as that found in conventional saccade adaptation between the magnitudes of saccade gain change in the adapt-up and adapt-down conditions (Figure 2). The adapt-down condition demonstrated a significant decrease while there was no significant change in the adapt-up condition in Experiment 1. Conventionally, subjects frequently do not increase gain and an asymmetry in the degree of adaptation between the two directions has often been observed (reviewed in [12, 52–54]). Therefore, it is unsurprising that we also did not find an increase in gain in the adapt-up salient distractor condition, possibly because the putative attentional error signal alone was too weak to induce saccade adaptation in a direction which is already difficult to adapt. One might even argue that the matching asymmetry in our data and in normal adaptation supports the relevance of our data to more standard saccade adaptation studies.

### 4.5. Neural Correlate of Saccade Adaptation

Further support for the interpretation of the current results in terms of attention induced adaptation can be found by considering the possible neural basis of the protocol. The neural correlate of saccade adaptation is thought to be either the superior colliculus (SC) or the cerebellum (reviewed in [12]). More recently, saccade adaptation, very similar in spatial and temporal dynamics to what can be produced by the McLaughlin method [6], was elicited by subthreshold microstimulation of the SC immediately after the saccade [55, 56]. Interestingly, it has also been found that subthreshold stimulation of the SC can cause shifts of attention [57, 58]. Hence, not only is it likely that the SC plays a major role in saccade adaptation, but also the SC has a role in attentional shifts. Therefore, the distractors in our study might have acted as bottom-up visual transients on an SC map, producing adaptation by the same mechanism as these two recent studies. Because the target was always present, competing with these transients, any such attentional error signal originating in the SC would be expected to result in smaller magnitude effects compared to conventional saccade adaptation.

In conclusion, by using salient visual distractors suddenly appearing after a saccade, the current results show that adaptation can be induced even in the presence of an unambiguous target location. This supports the notion that consistent differences between the locus of attention before and after a saccade may act as an error signal for saccade adaptation. Because the locus of attention is strongly influenced by predictive information [17], this novel hypothesis may provide an alternative interpretation for previous findings arguing for retinal or predictive error mechanisms of saccade adaptation.

## Figures and Tables

**Table 1:** Corrective saccades in Experiment  1. The percentage of adaptation trials with corrective saccades for the adapt-down and adapt-up condition, respectively, and the percentage of those that were in the direction of the distractor and that landed nearer the distractor compared to the saccade target.

Subject	Adapt-down condition			Adapt-up condition		
	Trials with corrective (%)	Trials with backward correctives (%)	Trials with correctives nearer distractor (%)	Trials with corrective (%)	Trials with forward corrective (%)	Trials with correctives nearer distractor (%)
S1	86.0	2.2	0.0	79.4	70.9	0.4
S2	87.2	1.1	0.0	40.6	37.6	2.1
S3	86.2	7.9	0.0	69.5	56.8	0.0
S4	76.8	5.2	2.4	84.1	80.2	4.0
S5	78.3	2.2	0.0	63.9	59.0	0.0
S6	84.3	6.6	5.4	86.6	86.1	12.4
S7	95.0	0.5	0.0	87.3	83.5	0.8
S8	90.1	0.9	0.9	93.5	92.9	11.8
S9	50.0	28.0	11.5	72.6	64.2	56.2
S10	13.5	5.7	0.0	36.6	19.8	0.0
S11	79.6	2.8	0.0	80.1	70.2	3.1
S12	49.3	2.8	0.9	58.4	57.9	5.6
S13	79.1	1.9	0.0	86.6	83.1	1.7
S14	75.8	3.1	0.6	77.4	74.9	4.1

Mean	73.7	5.1	1.6	72.6	66.9	7.3

Median	79.4	2.8	0.0	78.4	70.5	2.6

**Table 2:** Corrective saccades in Experiment  2. The percentage of adaptation trials with corrective saccades for the salient distractor and non salient distractor condition, respectively, and the percentage of those that were in the direction of the distractor and that landed nearer the distractor compared to the saccade target.

Subject	Salient distractor condition			Nonsalient distractor condition		
	Trials with corrective (%)	Trials with backward correctives (%)	Trials with correctives nearer distractor (%)	Trials with corrective (%)	Trials with backward corrective (%)	Trials with corrective nearer distractor (%)
S1	86.0	2.2	0.0	76.3	0.0	0.0
S2	87.2	1.1	0.0	24.4	0.0	0.0
S15	53.6	4.8	0.5	73.8	8.1	0.0
S16	27.6	9.9	1.6	53.0	22.1	1.1
S17	57.7	0.0	0.0	79.9	2.9	0.5
S18	63.1	3.6	1.2	72.7	11.0	0.5
S19	68.9	3.3	1.1	85.3	2.6	5.2
S20	77.7	15.5	1.5	82.2	21.2	0.5
S21	74.3	2.8	0.0	84.7	1.5	0.0
S22	86.1	5.6	2.8	73.2	9.5	0.6

Mean	68.2	4.9	0.9	70.6	7.9	0.8

Median	71.6	3.4	0.8	75.1	5.5	0.5
